# Genetic and Epigenetic Effects of Environmental Mutagens and Carcinogens

**DOI:** 10.1155/2015/608054

**Published:** 2015-08-04

**Authors:** Alessandra Pulliero, Jia Cao, Luciana dos Reis Vasques, Francesca Pacchierotti

**Affiliations:** ^1^Department of Health Sciences, University of Genoa, Via Antonio Pastore 1, 16132 Genoa, Italy; ^2^Toxicology Institute, Preventive Medical College, Third Military Medical University, Chongqing 400038, China; ^3^Department of Genetics and Evolutionary Biology, Institute of Bioscience, University of São Paulo, 05508-090 São Paulo, SP, Brazil; ^4^Laboratory of Toxicology UT-BIORAD, ENEA, CR Casaccia, Via Anguillarese 301, 00123 Rome, Italy

Identifying the effects of environmental exposures on human health is a major objective of life sciences and biomedical research. In environmental health, the recognition that exposures could produce DNA mutations represents a major landmark for risk assessment and prevention [1]. Consequently, chemical agents have been categorized according to their ability to alter the DNA sequence. Such information has been fundamental to determine environmental risks and shape current regulatory efforts for exposure reduction. Recent evidences suggest that the molecular influence of the environment may extend beyond the interaction with the DNA sequence [2]. Epigenetics is the study of heritable changes in gene expression that occur without changes in DNA sequence [3]. This is particularly fascinating because epigenetics involves factors that cause chemical changes to occur in our genomes. Epigenetic mechanisms, with particular reference to microRNA alterations, DNA methylation, and histone modifications, can change genome function under exogenous influence. MicroRNAs are posttranscriptional regulators of gene expression involved in carcinogenesis, metastasis, and invasion. Thus, microRNAs are potentially useful as diagnostic and prognostic biomarkers as well as anticancer therapeutic targets. Many microRNAs undergo altered expression as consequence of exposure to carcinogens and these changes may be useful in cancer prevention [4]. The relationship between exposure to environmental carcinogens and epigenetics revealed that toxicants modify epigenetic states. Accordingly, the alteration of microRNA expression is a general mechanism playing an important pathogenic role in linking exposure to environmental toxic agents with their pathological consequences. The underlying molecular mechanism involves both p53-microRNA interconnection and alterations of Dicer function [5]. MicroRNAs have been also implicated in transgenerational epigenetic inheritance via the gametes. Other epigenetic mechanisms, like DNA methylation, are susceptible to environmental influence in both somatic and germ cells modulating the mechanisms and the risk of chemically induced genetic and epigenetic effects. The present special issue provides a comprehensive overview of the current status of this interesting field of research. It comprises manuscripts reporting novel data as well as state-of-the-art reviews. We have composed a balanced issue combining in vitro studies, as well as studies performed in humans. The issue is started by a comprehensive review by M. Romani et al., describing the mechanisms of epigenetic control and their role in disease development, the influence of the environment, lifestyle, and nutritional habits influence on epigenetic markers, and how these factors are related to the development of cancer and other noncommunicable diseases. Diet is a major exposure daily route to many toxic substances including endocrine disruptors such as bisphenol A. The epigenome of mammalian female germ cells and oocyte development is modified by low bisphenol A concentrations with functional consequences on gene expression, chromosome dynamics in meiosis, and oocyte development. These important aspects are described by U. Eichenlaub-Ritter and F. Pacchierotti underlying that there are specific time windows, during which profound chromatin remodeling occurs and maternal imprints are established or protected that appear particularly vulnerable to epigenetic deregulation by bisphenol A. Indeed, the BPA exposure has long lasting consequences on the female reproductive health, reducing fertility and potentially leading to offspring defects. At the same time, certain components of the diet can modify the epigenetic pattern through natural bioactive components that can act on DNA methylation or histone modification or, as in the case of exogenous miRNA that can be direct epigenetic actors. Although much effort has focused on the gene-environment axis, there is a growing body of information suggesting that environmental influences extend beyond the DNA sequences of our genes. Epigenetics includes the study of the protein constituents of chromatin, the interaction of microRNAs with the genome, and the protein and DNA modifications that appear to define biologic states in local regions of chromosomes. However, only more recently the role of noncoding RNAs (ncRNAs) in epigenetic processes has been highlighted. This topic is discussed in P. Arrigo and A. Pulliero's paper dealing with the effect of environmental chemical stress on nuclear noncoding RNA involved in epigenetic control. This paper summarizes how a chemical mutagen can interfere with the conformation of specific regulatory domains in ncRNAs. The majority of human cellular RNA consists of rRNA (~90% of total RNA). The total number of RNA molecules is estimated to be about 10^7^ per cell, and ncRNAs include snRNA, snoRNA, and miRNA. The overexpression of lncRNAs can potentially measure the cytotoxicity signals of various environmental stresses [6]. Knowing the location of such ncRNAs could help in selecting the best candidates for starting the ncRNA-based gene therapy trials. For example, the fact that miRNA altered expression in cancer cells has a pathogenic effect provides the rationale for using miRNAs as potential therapeutic targets in cancer. It is interesting to note how the chemical entity could induce a slight change in the conformation that is critical for molecular recognition in the process of RNA editing. Environmental exposures to potentially aneugenic agents, including therapeutic exposures and exposures associated with lifestyle habits, influence the epigenetic alterations. In India, the health of approximately 62 million people is at risk due to high concentration of fluoride in drinking water/diet. The paper of A. P. Daiwile et al. describes the expression profiling of short noncoding RNAs, in particular miRNAs and snoRNAs, carried out in sodium fluoride (NaF) treated human osteosarcoma cells to understand their possible role in the development of fluorosis. They found that epigenetic modifications in miRNAs and C/D box snoRNAs that can regulate the key genes are involved in the development of fluorosis, suggesting the possible involvement of miR-124 and miR-155 in the development of fluorosis. The special issue is continued by three contributions describing mutations and epigenetic events important in our understanding of molecular mechanisms associated with exposure-disease outcomes. The review by O. Torres-Bugarin et al. provides strong information that sustains the observation that increased micronuclei frequencies are observed in association with the presence of any autoimmune disease condition. The authors propose that micronuclei could be used as an early biomarker of disease progression and/or response to therapy. The effect of toxicological agents on genes is an area that warrants further research as a major public health issue. In particular, pollution in Asia is a growing concern also in occupational places. Consequently, human molecular epidemiological studies have explored the association between polymorphisms of DNA repair genes and chromosomal damage by 1,3-butadiene in current exposure workers. The paper by M. Xiang et al. focuses on genetic polymorphisms of DNA repair genes and chromosomal damage. In their paper, they provide evidence for DNA repair genes (*XRCC1*,* MGMT*,* APE1*, and* ADPRT*) that modify the genetic instability induced by 1,3-butadiene, group 1 carcinogen, and widely used as an industrial chemical and also present in autoemission. Newly developed biomarkers (MNi: micronuclei; NPBs: nucleoplasmic bridges; NBUDs: nuclear buds) in the cytokinesis-blocked micronucleus cytome assay were used to detect chromosomal damage, showing that 1,3-butadiene exposed workers have increased frequencies of MNi and NPBs compared to control subjects. Actually, the effect on cell transformation of many environmental factors, which are reported in epidemiological studies to be associated with a certain risk for a given cancer type, might well be mediated by epimutations. The paper by S. A. Gross and K. M. Fedak nicely summarizes the role of such epimutations applying a weight of evidence approach to evaluate molecular characteristics for environmental carcinogens as well as associated disease outcomes. The authors consider the example of benzene as an environmental carcinogen and myelodysplastic syndrome as a causative disease endpoint. Using the weight of evidence method, they find overlapping polymorphisms in the metabolic enzymes GST and NQO1. Additional mutations including RUNX1 and glycophorin A, epigenetic events associated with DNMT3a, and polymorphism in interleukin cytokines induce dysregulation of the hematopoietic system in benzene-exposed workers. The weight of evidence approach illustrated by S. A. Gross and K. M. Fedak is a useful tool for sorting, categorizing, and prioritizing the most meaningful information. Using such a framework, researchers can determine ways to take advantage of best practices in identifying exposure scenarios and defining biomarkers relevant to the exposure-disease paradigm. In addition, certain toxic metals (i.e., cadmium, nickel, arsenic, and chromium) have been identified as persistent environmental pollutants due to their indestructible chemical and physical properties. While the ability of some metal compounds to cause cancer in exposed workers has been known for a long time, epigenetic-based research has recently shed light upon mechanisms involved in metal-induced carcinogenesis [7]. For example, recent research has shown that cadmium exposure stimulates cellular proliferation, increases oxidative stress and DNA damage, and induces global DNA hypomethylation [8]. C. Urani et al., in the experimental study, provide new information on the novel mechanisms for cadmium toxicity and biological effects and, along with those already reported in the literature, open new intriguing interpretations on cadmium-induced carcinogenicity. The authors show that HepG2 cells, exposed to 10 *μ*M cadmium for 24 hrs, increase microarray expression profiling of labile zinc after cadmium exposure. In addition, the downregulation of miR-34a and miR-200a, both implicated in the epithelial-mesenchymal transition, supports the role played by zinc in affecting gene expression at the posttranscriptional level. Research on epimutations is also a rapidly growing area. These changes may involve modification of DNA bases and histones, such as methylation, ubiquitination, or acetylation. The regulation of transcription and genome stability by epigenetic systems is crucial for the proper development of mammalian embryos. Y. Arai et al. confirm the hypothesis that combined exposure to chemicals (diethyl phosphate, mercury, cotinine, selenium, and octachlorodipropyl ether) detected at low concentrations in maternal peripheral and cord blood samples affect embryoid body formation and neural differentiation from human induced pluripotent stem cells (hiPSCs). C. E. Cross et al. add another detail reporting that short-term exposure of villous first trimester trophoblasts to low concentrations of H_2_O_2_ significantly alters miRNA profile and expression of genes implicated in placental development. The last section of this special issue comprises two papers describing the features of DNA methylation and the exposure to a variety of chemical stressors, occurring during the prenatal or the adult life. The first paper by F. Pacchierotti and M. Spanò reviews the literature on the impact of chemicals and lifestyle factors on DNA methylation in germ cells. Studies on rodents show that DNA methylation can be altered by many different kinds of exposures during the fetal as well as the adult life. Still these studies suffer from some limitations: few experiments were conducted on the female germline; the analyses were rarely extended to a genomewide scale; only in a few cases the functional impact of epigenetic changes was investigated; dose-response relationships were scarcely considered. Nevertheless, their results are very important because they establish proof of principle demonstration that a variety of exogenous stressors may alter DNA methylation at developmentally important imprinted or metabolic genes and warrant future investigations on human mother-child cohorts and biomonitoring epigenetic analyses on semen collected from human exposed cohorts. In a final paper, Y. Liu et al. demonstrated for the first time that 50 Hz ELF-EMF exposure can induce the alterations of genomewide methylation and the expression of* DNMTs *in spermatocyte-derived GC-2 cells. Indeed, the epigenetics plays an important role in the biological effects of 50 Hz ELF-EMF exposure.

Understanding the mechanistic basis of how epigenetic regulation is achieved is fundamental to placing this level of biologic regulation in the toolbox of environmental health science and medicine. Based on these contributions, it may be stated that the genetic and epigenetic effects of environmental mutagens and carcinogens are subject of innovative research.
